# Epidemiologic Features and Control Measures during Monkeypox Outbreak, Spain, June 2022

**DOI:** 10.3201/eid2809.221051

**Published:** 2022-09

**Authors:** Berta Suárez Rodríguez, Bernardo R. Guzmán Herrador, Asunción Díaz Franco, María Paz Sánchez-Seco Fariñas, Julia del Amo Valero, Adrián Hugo Aginagalde Llorente, Juan Pablo Alonso Pérez de Agreda, Rosa Carbó Malonda, Daniel Castrillejo, María Dolores Chirlaque López, Eduardo Javier Chong Chong, Sonia Fernández Balbuena, Virtudes Gallardo García, Manuel García-Cenoz, Laura García Hernández, Elisa Gil Montalbán, Fernando González Carril, Teresa González Cortijo, Susana Jiménez Bueno, Aurora Limia Sánchez, Juan Antonio Linares Dópido, Nicola Lorusso, Mario Margolles Martins, Eva María Martínez Ochoa, Ana Martínez Mateo, Jacobo Mendioroz Peña, Ana Isabel Negredo Antón, María Teresa Otero Barrós, Maria del Carmen Pacheco Martinez, Pilar Peces Jiménez, Oscar-Guillermo Pérez Martín, Ana Isabel Rivas Pérez, María Sastre García, National Monkeypox Response Group, Fernando Simón Soria, María José Sierra Moros

**Affiliations:** Coordinating Centre for Health Alerts and Emergencies, Directorate General of Public Health, Ministry of Health, Madrid, Spain (B. Suarez Rodriguez, B.R. Guzmán Herrador, E.J. Chong Chong, S. Fernández Balbuena, F. Simón Soria, M.J. Sierra Moros);; National Centre of Epidemiology, Carlos III Health Institute, Madrid (A. Díaz Franco, M. Sastre García);; National Centre for Microbiology, Instituto de Salud Carlos III, Madrid (M.P. Sánchez-Seco Fariñas, A. I. Negredo Antón);; Division for HIV Control, STI, Viral Hepatitis and Tuberculosis, Ministry of Health, Madrid (J. del Amo Valero);; Public Health Observatory of Cantabria, Cantabria, Spain (A.H. Aginagalde Llorente);; Dirección General de Salud Pública, Aragón, Spain (J.P. Alonso Pérez de Agreda);; Subdirecció General d’Epidemiologia, Vigilància de la Salut i Sanitat Ambiental, Comunidad Valenciana, Spain (R. Carbó Malonda);; Consejería de Políticas Sociales, Salud Pública y Bienestar Animal, Melilla, Spain (D. Castrillejo);; Regional Health Council, IMIB-Arrixaca, Murcia University, Murcia, Spain (M.D. Chirlaque López);; Ministry of Health and Families of Andalusia, Andalusia, Spain (V. Gallardo García, N Lorusso);; Instituto de Salud Pública de Navarra, Spain and Navarre Institute for Health Research (IdiSNA), Pamplona, Spain (M. García-Cenoz);; Dirección General de Salud Pública del Servicio Canario de la Salud, Tenerife, Spain (L. García Hernández, O.-G. Pérez Martín);; Dirección General de Salud Pública de la Comunidad de Madrid, Madrid (E. Gil Montalbán, S. Jiménez Bueno);; Departamento de Salud del País, Vasco, Spain (F. González Carril);; Dirección General de Salud Pública, Islas Baleares, Spain (T. González Cortijo);; Immunization Programme Area, Directorate General of Public Health, Ministry of Health, Madrid (A. Limia Sánchez);; Dirección General de Salud Pública. Servicio Extremeño de Salud, Merida, Spain (J.A. Linares Dópido);; Dirección General de Salud Pública. Gobierno de Asturias, Oviedo, Spain (M. Margolles Martins);; Dirección General de Salud Pública, Consumo y Cuidados de la Consejería de Salud de La Rioja, Logroño, Spain (E.M. Martínez Ochoa);; Public Health Agency of Catalonia, Barcelona, Spain (A. Martinez Mateo, J. Mendioroz Peña);; Dirección Xeral de Saúde Pública, Consellería de Sanidade, Xunta de Galicia, Santiago, Spain (M.T. Otero Barrós);; Dirección General de Salud Pública, Castilla y León, Valladolid, Spain (M.C. Pacheco Martinez);; Servicio de Epidemiología, Castilla-La Mancha, Toledo, Spain (P. Peces Jiménez);; Consejería de Sanidad, Consumo y Gobernación. Ciudad Autónoma de Ceuta, Ceuta, Spain (A.I. Rivas Pérez);; CIBER in Infectious Diseases, Madrid, CIBERINFEC (M.P. Sánchez-Seco Fariñas, A.I. Negredo Antón, M.J. Sierra Moros, A. Díaz Franco);; CIBER in Epidemiology and Public Health, Madrid, CIBERESP (M.D. Chirlaque López, M. García-Cenoz, A. Martinez Mateo, M. Sastre García, F. Simón Soria)

**Keywords:** monkeypox, outbreak, multicountry, response, monkeypox viruses, viruses, epidemiologic features, control measures, men who have had sex with men, MSM, bioterrorism and preparedness, zoonoses, Spain

## Abstract

During June 2022, Spain was one of the countries most affected worldwide by a multicountry monkeypox outbreak with chains of transmission without identified links to disease-endemic countries. We provide epidemiologic features of cases reported in Spain and the coordinated measures taken to respond to this outbreak.

During May‒June 2022, after an alert notification initiated by the United Kingdom (*1**,**2*), >4,500 monkeypox cases had been confirmed worldwide, mainly in the European region (*3**–**6*). Chains of transmission without links to disease-endemic countries have been identified, and cases have occurred mainly among men who have had sex with men (MSM) in high-risk sexual contexts (*5**,**6*).

During June 2022, Spain was one of the countries most affected by monkeypox. We provide epidemiologic features of monkeypox cases reported to the National Surveillance Network through July 4, 2022 (*7*), complemented with information obtained from bilateral consultations with the Spanish Autonomous Regions, and the measures taken to respond to this alert. No ethics approval was sought because this study describes cases and public health actions in Spain linked to the ongoing multicounty outbreak during June 2022. No personal identifiable data for case-patients or any contacts are included in this report.

## The Study

Suspected monkeypox cases in Spain were initially reported on May 17. By July 4, of the 19 Autonomous Regions in Spain, 16 had reported 1,256 cases, of which 61.1% (n = 768) were reported by the Region of Madrid. A total of 1,242 cases were in men and 14 in women. The median age of case-patients was 37 years; all but 1 case-patients were adults (Table).

**Table:** Characteristics of 1,256 monkeypox case-patients, Spain, July 4, 2022

Characteristic	No. (%) case-patients
Sex	
M	1,242 (98.9)
F	14 (1.10
Age group, y	
<20	6 (0.5)
20‒39	238 (18.9)
30‒39	511 (40.7)
40‒49	361 (28.7)
50‒59	126 (10.0)
>60	13 (1.0)
Unknown	1 (0.1)
General symptoms, n = 530*	
General	
Fever	302 (56.9)
Asthenia	224 (42.3)
Muscle pain	167 (31.5)
Throat pain	136 (25.7)
Headache	140 (26.4)
Specific	
Anogenital rash	355 (66.9)
Disseminated rash in locations other than anogenital or oro/peribuccal	293 (55.3)
Localized lymphadenopathy	216 (40.7)
Oro/peribuccal rash	92 (17.4)
General lymphadenopathy	45 (8.5)

*Each case-patient can have >1 symptom.

Date of symptom onset was known for 1,182 (89.5%) case-patients . An epidemic plot showed a sustained increasing trend during May and June (Figure). The decreasing numbers during the second half of June might be caused by a delay in reporting.

**Figure:**
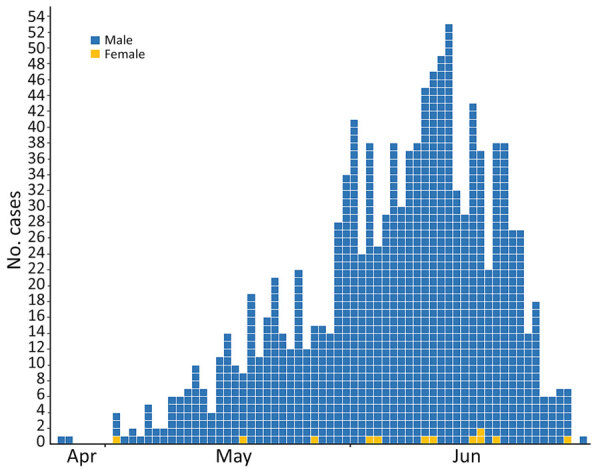
Epidemic plot of 1,182 confirmed cases of monkeypox according to date of onset of symptoms for late April, May, and June 2022, Spain. April cases were reported during April 25–30. Cases without date of symptom onset were not included (for 72 male case-patients and 2 female case-patients).

We obtained information from 4 different series from 4 regions involving 45 patients who self-referred a clear exposure date (range 4‒22 patients/region). This information showed average incubation periods of 7‒9.6 days.

The most frequent symptoms reported (n = 530) were rash (mainly anogenital), fever, asthenia, and lymphadenopathy (Table). Most patients had >1 general symptom plus disseminated and anogenital rash (126 case-patients), >1 general symptom plus anogenital rash (exclusively) (105 case-patients), or >1 general symptom plus diseminated rash (without anogenital or oro/peribuccal location) (76 case-patients). Of the 216 case-patients who had localized lymphadenopathy, 191 had general symptoms. The median number of days from symptom onset to rash was *<*1 day (IQR 0–2 days; information was available for 427 case-patients). A total of 30 of the 530 case-patients were hospitalized (median admission 2 days); 33 reported complications, mainly secondary bacterial infections (n = 15) oral ulcers (n = 11), proctitis (n = 2), and pharyngotonsillitis (n = 2). No deaths were reported.

Of 440 case-patients who had available information, 62 had traveled to countries that had reported monkeypox cases during the incubation period. A total of 101 case-patients were reported to be close contacts of confirmed or probable case-patients.

The most likely mechanism of transmission reported by 332 (85.8%) of the 387 case-patients who had available information was intimate and prolonged contact during sex. A total of 31 case-patients reported close contacts unrelated to sex; for 24 case-patients, this information was pending. Of those 332 case-patients, 290 were MSM; 6 reported heterosexual contact, and information was pending for the remaining 36. Of 413 case-patients who had available information, 163 had attended a mass gathering before symptom onset; 101 attended Pride events in different cities in Spain. Regarding the 14 women, 7 reported intimate contact during sex with men and 2 had close contacts within the family environment; for 5 women, this information was pending.

A total of 11 regions representing 73% of the case-patients reported provided additional details on contact tracing. It was not feasible to identify or obtain any information regarding potential contacts for a substantial number of patients. Most regions reported an average of <3 identifiable contacts/case-patient. Only 4 regions reported case-patients that had >5 identifiable contacts.

The case definition for monkeypox in Spain considers a confirmed case-patient as a person who had monkeypox genome identified by PCR or who had a positive result in a generic PCR for *Orthopoxvirus* in a clinical sample. However, the first confirmation of monkeypox cases was conducted by using sequencing (*8*).

The National Centre for Microbiology conducted partial sequencing of 23 patients and the complete sequencing in samples from 24 cases. This testing identified the West African clade of monkeypox virus.

Following the procedures of the National Early Warning and Rapid Response System, all key stakeholders were alerted to pursue a rapid and coordinated response. A national protocol for early detection and case and contact management was approved and made available by the National Alert Board (*8*) and coordinated by the Ministry of Health 3 days after detection of suspected cases. A rapid risk assessment for Spain has been reported (*9*), and situation reports are updated regularly (*3*). Early consultation and exchange with relevant scientific societies led to publication of an atlas that contained differential diagnoses for monkeypox skin lesions (*10*).

Partnership with the lesbian, gay, transgender, bisexual, intersex, and queer (LGTBIQ) community was seen as pivotal, and the Ministry of Health involved its Advisory and Counselling Board of nongovernmental organizations in the response to promote the engagement of the LGTBIQ community. In this context, several materials, with key health messages developed, are publicly available (*11*), building on previous experience on safe sex campaigns, during summer events and following the general principles of the World Health Organization and European Centre for Disease Prevention and Control (*12*).

Recommendations to offer monkeypox vaccine as postexposure prophylaxis to close contacts, especially those at high risk of developing severe disease and healthcare workers experiencing incidences with the personal protective equipment when in contact with patients, have been proposed by the National Board for Vaccines (*13*), and >80 contacts (information available from 12 regions) have already been vaccinated. Use of vaccination as preexposure prophylaxis for high-risk groups and healthcare workers with occupational risk is now under discussion as the availability of vaccine increases.

## Conclusions

Monkeypox transmission is currently centered, but not exclusively, in MSM who have close physical contact in high-risk sexual contexts. However, without optimal control, there is a risk for transmission to other population groups. Early detection, which requires useful information for the differential diagnosis of clinical manifestations, is crucial to control transmission, as is timely case reporting. It is also essential to continue characterizing the dynamics of the outbreak to identify potential changes to tailor and adapt recommendations.

One of the main challenges encountered in the response to this alert is identifying and tracking contacts: case-patients might be hesitant to provide the identities of their contacts or might not be able to do so because risk exposures had occurred anonymously with previously unknown persons. In certain occasions, it was also difficult to ascertain during the epidemiologic interview the exact date in which transmission might had occurred.

The way the ongoing monkeypox outbreak will evolve is still uncertain and will be influenced by how successfully advice reaches the population at risk. Effective risk communication and community engagement strategies are paramount to delivering information to the general population and to most at-risk persons, including summer mass-gathering event organizers. These features should include clear and contrasted information in partnership with the LGTBIQ community to minimize risk behaviors and maximize awareness about the importance of following public health control measures. Explicit warnings to avoid any form of stigmatizing the LGTBIQ community should frame all interventions.
